# Recovery of metagenome-assembled genomes from *Spartina alterniflora* root microbiome in Fujian Province, China

**DOI:** 10.1038/s41597-026-06914-z

**Published:** 2026-02-26

**Authors:** Zhaobin Huang, Jillian M. Petersen

**Affiliations:** 1 College of Oceanology and Food Science, Quanzhou Normal University, Quanzhou, PR China; 2 Fujian Province Key Laboratory for the Development of Bioactive Material from Marine Algae, Quanzhou, PR China; 3 Centre for Microbiology and Environmental Systems Science, University of Vienna, Vienna, Austria; 4 Vienna Doctoral School of Microbiology and Environmental Science, University of Vienna, Vienna, Austria; 5 Environment and Climate Hub, University of Vienna, Vienna, Austria

**Keywords:** Soil microbiology, Microbial ecology

## Abstract

The saltmarsh cordgrass *Spartina alterniflora* proliferates along the coast of China. Like all plants, *S. alterniflora* hosts a specific microbiome that plays crucial roles in sustaining plant growth and health. Till now, very few studies have investigated the root microbiome of *S. alterniflora* in China, where it is considered an invasive pest. Here, ~350 Gbp metagenomes of *S. alterniflora* were generated from 8 sampling sites in South Fujian Province, China. 798 bacterial metagenome-assembled genomes (MAGs) and 7 archaeal MAGs were obtained, which were de-replicated into 205 and 3 representative genomes at a 95% ANI cutoff. The recovered bacterial MAGs mainly belonged to Gammaproteobacteria, Alphaproteobacteria, Bacteroidia and Campylobacterota. Sedimenticolaceae were prevalent at all sampling sites, accounting for 4–30% of the corresponding MAGs. These genomic datasets provide a new resource for investigating *S. alterniflora* root microbiomes, particularly valuable considering current efforts to eradicate this species in China.

## Background & Summary

The saltmarsh cordgrass *Spartina alterniflora*, an invasive plant species (also called *Sporobolus alterniflorus*^1^) has flourished along the coast of China from the north to south since its introduction in 1979^2,3^. The coasts of two estuaries, Jinjiang and Luoyangjiang, which flow into Quanzhou Bay, South Fujian Province, China, became densely covered by the cordgrass *S. alterniflora* (Figure S1a). However, due to its negative impact on the coastal ecosystems, China launched the *Spartina* removal project to remove cordgrass along the coastline, including Quanzhou Bay^4^, starting in February 2023.

It is now well established that microorganisms associated with plant roots, the ‘plant root microbiome’, play crucial roles in sustaining plant growth and health^5^, and in facilitating the resistance to the changes posed by environmental stress, such as sulfide toxicity^6,7^ and salinity^8^. Cordgrass samples that had a well-developed root with a dense short- and thread-shaped root hair were collected in Quanzhou Bay (Figure S1b), to investigate the phylogenetic and genomic diversity of the cordgrass root microbiome in these invasive settings. Recent studies found that the diazotrophic and sulfur-oxidizing chemolithoautotrophic Sedimenticolaceae bacteria dominated root microbiomes of *S. alterniflora* thriving along the United States Atlantic and Gulf of Mexico coastlines^9,10^, which is the native range for this plant. Sedimenticolaceae, a family within the Gammaproteobacteria that also contains the chemolithoautotrophic symbionts of lucinid clams, many of which are capable of nitrogen fixation^11,12^, have been hypothesized to contribute a nitrogen source for the cordgrass^9^. It is so far unknown where this symbiosis exists in *S. alterniflora* in invasive settings, such as along the coast of China. Like most environmentally relevant microorganisms, there are very few cultured representatives of this group^13,14^. Advances in sequencing technologies and bioinformatic tools for assembly and genome binning now make it possible to investigate the genomic potential of these and other cordgrass-associated microorganisms independent of the ability to culture them.

**Fig. 1:**
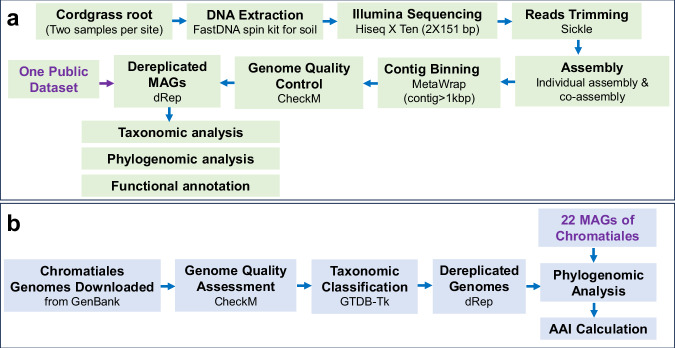
Overview of the metagenomic analysis of *S. alterniflora* root microbiome. (**a**) Pipeline of the data processing from samples to the assembled MAGs. (**b**) Pipeline of the phylogenomic analysis of the MAGs assigned to the family Sedimenticolaceae.

**Fig. 2:**
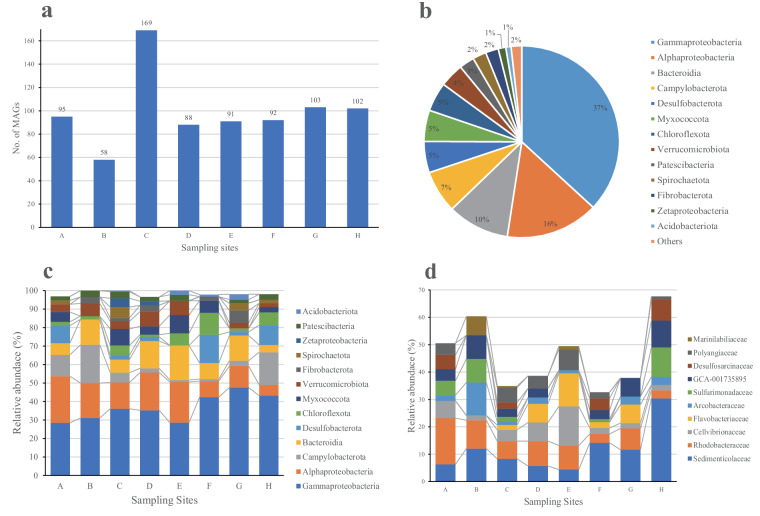
Overview of the metagenome-assembled genomes (MAGs) from the cordgrass root microbiome. (**a**) The number of MAGs obtained from each sampling site. (**b**) Classification of the MAGs. (**c**) Composition of MAGs in each sampling site classified at the class level. (**d**) Composition of MAGs in each sampling site classified at the family level.

In this study, metagenome-assembled genomes (MAGs) were produced from samples of the saltmarsh cordgrass *S. alterniflora* roots, collected from 7 sampling sites (named A-G) alongside the two estuaries to Quanzhou Bay coast, and one sampling site (named H) in Jiulongjiang estuary, Xiamen Bay (Figure S2). A summary of the main features of metagenomic datasets is presented in Supplementary Table S1. A total of 349.57 Gb paired end reads for the 16 samples (2 samples per each site), with an average of 21.85 Gb data per sample was retrieved. Through assembly and binning pipeline (Fig. 1a), a total of 805 metagenome assembled genomes (MAGs), including 798 bacterial MAGs and 7 archaeal MAGs was obtained (Table S2). All MAGs had completeness > 50% and contamination < 10% with a contig length > 1000 bp. Of the 798 bacterial MAGs, 306 MAGs were high quality (completeness > 90%, contamination < 5%), and the rest were medium quality (completeness > 50%, contamination < 10%), according to the Minimum Information about a Metagenomic Sequence (MIMS)^15^. A larger number of bacterial MAGs were obtained from sampling site C (n = 169) and a smaller number from sampling site B (n = 58) (Fig. 2a). The majority of the MAGs was found to belong to Gammaproteobacteria (n = 295; 37% of all bacterial MAGs), Alphaproteobacteria (n = 123; 15%), Bacteroidia (n = 82; 10%), Campylobacterota (n = 57; 7%), Desulfobacterota (n = 42; 5%), Myxococcota (n = 42; 5%), and Chloroflexota (n = 39; 5%) (Fig. 2b). At each site, Gammaprotoebacteria was the dominant group (Fig. 2c). MAGs affiliated to the family Sedimenticolaceae were detected at every site, accounting for 4–30% of all bacterial MAGs at each site. This indicated that Sedimenticolaceae was prevalent and widespread members of the cordgrass *S. alterniflora* root-associated microbiome in China. Several other families such as Rhodobacteraceae, Cellvibrionaceae, and ‘GCA-001735895’, were widely detected in the root microbiomes (Fig. 2d).

**Fig. 3:**
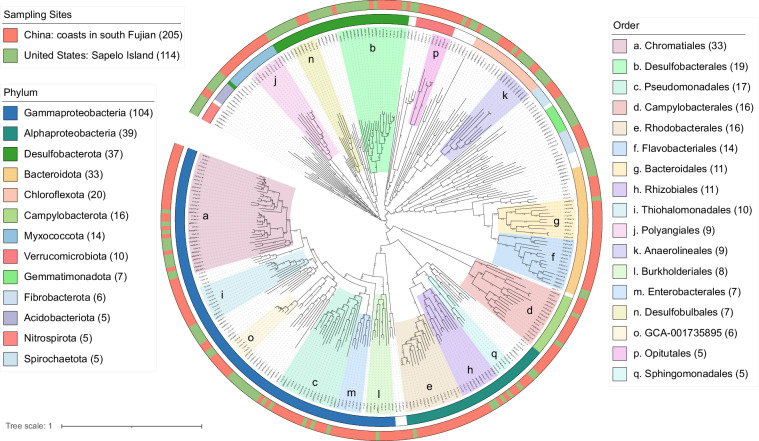
Phylogenomic tree based on the Bac120 of the representative MAGs. This analysis includes 205 bacterial MAGs obtain from cordgrass root microbiomes from this study, and 114 MAGs from cordgrass root, rhizosphere and sediment metagenomes published by Rolando *et al*.^9^. Bootstrap values > 70% are shown above the tree branches.

All MAGs were grouped into 205 unique bacterial species-level genomes and 3 archaeal genomes using dereplication at an average nucleotide identity (ANI) cutoff of 95%. We compared these with the representative MAGs obtained from the sediment, rhizosphere, and root samples of cordgrass *S. alterniflora* in Sapelo island in United States (n = 114)^9^, confirming that Gammaproteobacteria (n = 104) was the most dominant group associated with *S. alterniflora* at both locations. Within this taxonomic group, the most commonly represented orders were Chromatiales (n = 33, 22 MAGs from this study and 11 MAGs from the public dataset^9^), followed by Desulfobacterales (n = 19), and Pseudomonadales (n = 17) (Fig. 3).

**Fig. 4:**
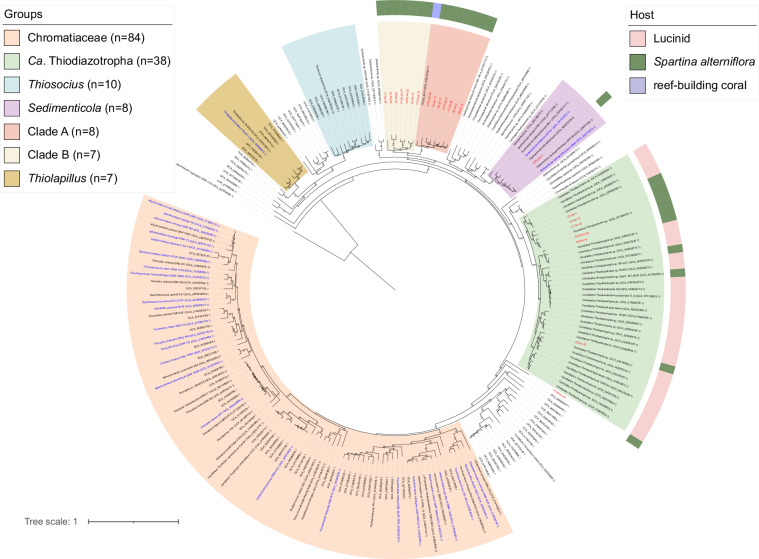
Phylogenomic tree based on the Bac120 of the order *Chromatiales*. MAGs obtained in this study are shown in red text. The host information is retrieved from GenBank.

To further investigate the Chromatiales MAGs, we constructed phylogenomic tree based on the Bac120 dataset^16^. Six representative MAGs from this study that were included in this analysis were assigned to the genus *Candidatus* Thiodiazotropha (Fig. 4). These six were not only tightly clustered with the MAGs associated with *S. alterniflora* in Sapelo Island, United State^9^, but also with the chemolithoautotrophic endosymbionts of marine lucinid clams^17^. *Ca*. Thiodiazotropha from distinct plant and animal hosts are intermixed, thus, transitions between these two host types have likely occurred during the evolution of this symbiont group (Fig. 4). Comparisons with the genomes of their closest animal-associated relatives may provide valuable insights into the functions and mechanisms needed for association with plant vs animal hosts by these bacteria.

Although most of our MAGs from Chromatiales affiliated with the genus *Ca*. Thiodiazotropha, one MAG formed a clade affiliated to the genus *Sedimenticola*, and 14 MAGs did not group together with previously published genomes (Fig. 4). These may present two potentially novel genera within the family Sedimenticolaceae (Fig. 4). So, to further confirm taxonomic novelty, we calculated the average amino acid identity (AAI) among the 14 representative MAGs (Figure S3). The 14 MAGs separated into two groups that had within-group AAI values of 70.2–91.9% and 75.0–86.6%, respectively. The genomes (n = 38) in the genus Ca. *Thiodiazotropha* shared > 70.8% AAI values among the representative MAGs from this study and public reference genomes (Figure S3).

Our study is the first report of the genomic potential of microbiome members from the saltmarsh cordgrass *S. alterniflora* root in invasive estuarine coastal settings in China. We have doubled the number of medium-to-high quality MAGs of root-associated microorganisms for this widespread plant. The genomes will provide valuable insights into bacterial diversity, functional potential, and the evolution of host-symbiont associations and guide us for further attempts to cultivate these important bacterial symbionts.

## Materials and Methods

### Sampling of the cordgrass *S. alterniflora*

Established plants of the cordgrass *S. alterniflora* were sampled from 7 sites numbered A, B, C, D, E, F, and G alongside the Jinjiang and Luoyangjiang coast (Quanzhou Bay) and one site numbered H alongside the Jiulongjiang estuary to Xiamen coast, South Fujian Province, from July 2023 to June 2024 (Figure S2 and Table S1). The cordgrass was pulled out from the sediment using a small shovel, and placed in a plastic bag, and taken to laboratory immediately. The root of the cordgrass was washed using seawater to remove clay, then root pieces (<9 cm in length) were cut off with scissors and put into a sterile petri dish. The root was then washed two or three times using sterile seawater and placed into a pre-prepared FastDNA spin kit lysis tube (MP Biomedicals, USA).

### DNA extraction, metagenomic sequencing, assembly and binning

Total DNA of the root microbiome was extracted using the FastDNA spin kit for soil following the manufacturer’s instructions. The DNA quality was checked using 1.0% agarose gel electrophoresis. DNA was fragmented and subjected to Illumina Hiseq Sequencing (MajorBio Pharm. Co., Shanghai, China). The paired-end (PE) reads were quality checked using sickle (https://github.com/najoshi/sickle) with the parameters (q = 20 and l = 50), and clean PE reads were assembled into contiguous sequences (contigs) using megahit v 1.2.9^18^. Reads were mapped to the available *S. alterniflora* genome (GenBank assembly accession GCA_008808055.3) with bowtie2 v 2.5.4^19^ to calculate the host DNA contamination. Binning of the contigs was performed using MetaWrap software^20^. The MAGs were dereplicated using dRep v3.4.3^21^ based on the ANI of 95%, and taxonomically classified using GTDB-Tk (Fig. 1a). The tree was viewed and annotated using iToL online^22^.

### Phylogenomic analysis of representative MAGs assigned to the order Chromatiales

To further refine the taxonomic placement of representative MAGs assigned to the order Chromatiales (Fig. 1b), we constructed a phylogenomic tree based on the bac120 dataset using GTDB-Tk v1.3.0^16^. Briefly, the Chromatiales reference genomes available till now were downloaded from GenBank (https://www.ncbi.nlm.nih.gov/datasets/genome/?taxon=135613, accessed on 13/05/2025), and genome quality was assessed using CheckM v 1.2.0 with completeness >90% and contamination <5%^23^. The taxonomic assignment of these genomes to the order Chromatiales was performed using GTDB-Tk v1.3.0^16^, and the genomes were dereplicated using dRep v3.4.3^21^. A phylogenomic tree was constructed using GTDB-Tk v1.3.0. The average amino acid identity of the related genomes was calculated using CompareM v0.1.2 (https://github.com/dparks1134/CompareM).

## Data Records

The information for all samples, including sampling data, statistics of the Illumina PE reads, and the assembled contigs are provided in Table S1. The raw reads generated for 16 metagenomes are available at NCBI Sequence Read Archive SRA under the accession number SRP648702^24^. The nucleotide sequences of each metagenome-assembled genome are provided in Table S2 and deposited in the GenBank under the BioProject accession number PRJNA1177211^25^.

## Technical Validation

Total DNA extracted from the root tissue of *S. alterniflora* was examined by 1.0% agarose electrophoresis and quantified using a NanoDrop 1000 spectrophotomer (ThermoFisher Scientific, USA).

## Supplementary information

Supplementary Information

Table S1

Table S2

## Data Availability

The raw reads for 16 metagenomes are available at NCBI Sequence Read Archive (SRA) under SRP648702. The MAGs have been deposited at DDBJ/ENA/GenBank under PRJNA1177211 and the accession numbers are also provided in Table S2.

## Code availability

The programs of the analysis are described in the main text, and available on GitHub: https://github.com/Zhaobin85/Metagenomic-Analysis.
