# Supplementary material for: Recovery of metagenome-assembled genomes from Spartina alterniflora root microbiome in Fujian Province, China
Source: Sci Data. 2026 Feb 26;13:541. doi: 10.1038/s41597-026-06914-z (PMC13061883; doi:10.1038/s41597-026-06914-z)
Supplement: Supplementary file 1 — Supplementary Information [file 41597_2026_6914_MOESM1_ESM.pdf]

Supplementary Information for

Recovery of metagenome-assembled genomes from *Spartina alterniflora* root microbiome in Fujian Province, China

Zhaobin Huang and Jillian M. Petersen

This PDF file includes: Fig. S1, Fig. S2 and Fig. S3.

The other supplementary information for this manuscript includes:

Table S1 and Table S2.

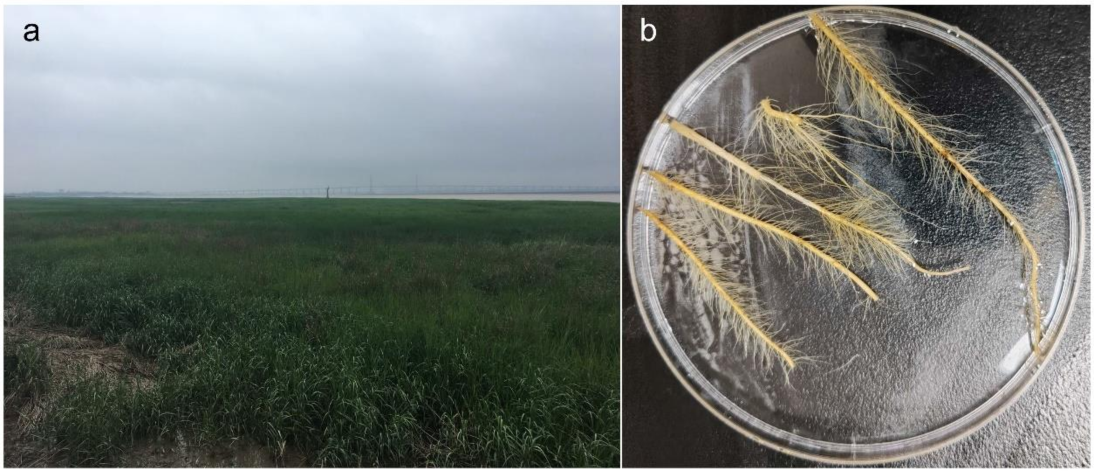

Fig. S1. The saltmarsh cordgrass *Spartina alterniflora* along the Quanzhou Bay coast and the morphology of root. (a) The left picture was taken in June 2019, and since the *S. alterniflora* removal project started in Feb. 2023, it was gone. (b) The root with a dense short- and thread-shaped hair.

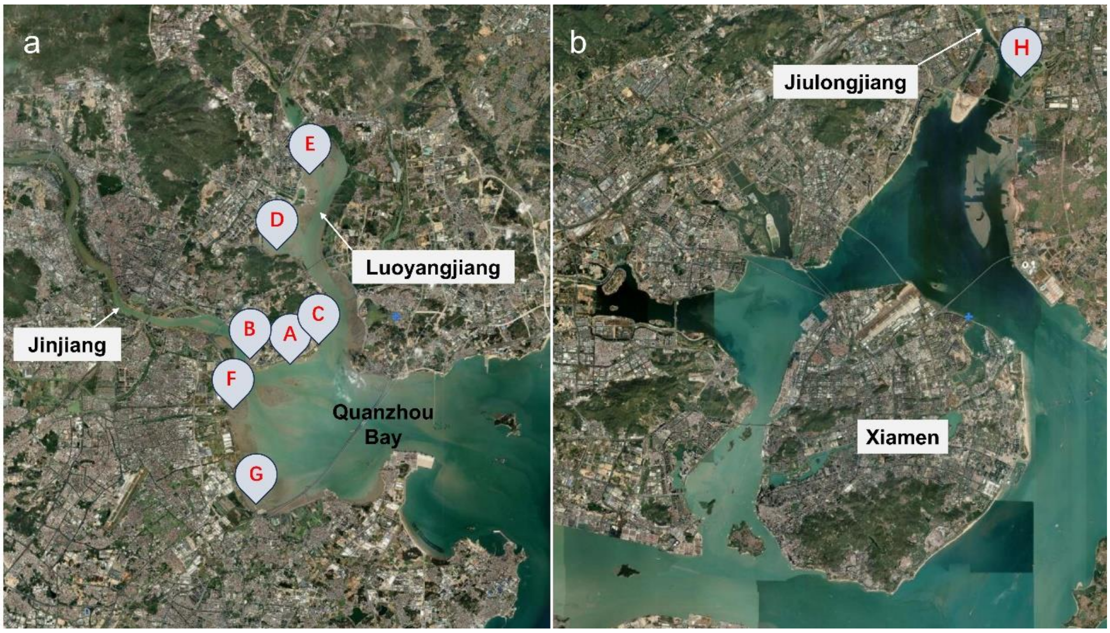

Fig. S2. Sampling sites viewed from the satellite. (a)The two estuaries, Jinjiang and Luoyangjiang, flow into Quanzhou Bay. (b) The Jiulongjiang estuary flows into Xiamen Bay.

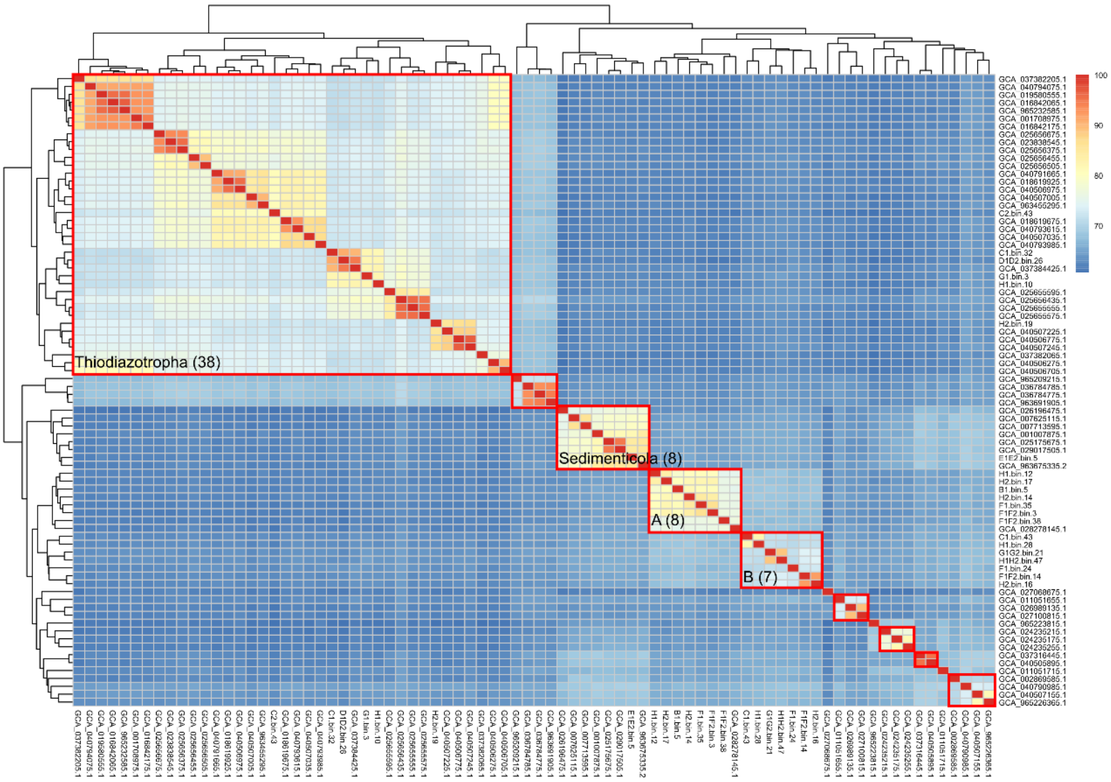

Fig. S3. Heatmap of AAI values calculated among the genomes of the family Sedimenticolaceae.
